# Quantifying intrafractional gastric motion using auto‐segmentation on MRI: Deformation and respiratory‐induced displacement compared

**DOI:** 10.1002/acm2.13864

**Published:** 2022-12-24

**Authors:** Theo Driever, Maarten C.C.M. Hulshof, Arjan Bel, Jan‐Jakob Sonke, Astrid van der Horst

**Affiliations:** ^1^ Department of Radiation Oncology The Netherlands Cancer Institute Amsterdam The Netherlands; ^2^ Department of Radiation Oncology Amsterdam UMC University of Amsterdam Amsterdam The Netherlands

**Keywords:** deformation, gastric cancer, intrafractional motion, radiotherapy

## Abstract

**Background and purpose:**

For accurate pre‐operative gastric radiotherapy, intrafractional changes must be taken into account. The aim of this study is to quantify local gastric deformations and compare these deformations with respiratory‐induced displacement.

**Materials and methods:**

Coronal 2D MRI scans (15–16 min; 120 repetitions of 25–27 interleaved slices) were obtained for 18 healthy volunteers. A deep‐learning network was used to auto‐segment the stomach. To separate out respiratory‐induced displacements, auto‐segmentations were rigidly shifted in superior‐inferior (SI) direction to align the centre of mass (CoM) within every slice. From these shifted auto‐segmentations, 3D iso‐probability surfaces (isosurfaces) were established: a reference surface for P_Occ_ = 0.50 and 50 other isosurfaces (from P_Occ_ = 0.01 to 0.99), with P_Occ_ indicating the probability of occupation by the stomach. For each point on the reference surface, distances to all isosurfaces were determined and a cumulative Gaussian was fitted to this probability‐distance dataset to obtain a standard deviation (SD_deform_) expressing local deformation. For each volunteer, we determined median and 98^th^ percentile of SD_deform_ over the reference surface and compared these with the respiratory‐induced displacement SD_resp_, that is, the SD of all CoM shifts (paired Wilcoxon signed‐rank, α = 0.05).

**Results:**

Larger deformations were mostly seen in the antrum and pyloric region. Median SD_deform_ (range, 2.0–2.9 mm) was smaller than SD_resp_ (2.7–8.8 mm) for each volunteer (*p* < 0.00001); 98^th^ percentile of SD_deform_ (3.2–7.3 mm) did not significantly differ from SD_resp_ (*p* = 0.13).

**Conclusion:**

Locally, gastric deformations can be large. Overall, however, these deformations are limited compared to respiratory‐induced displacement. Therefore, unless respiratory motion is considerably reduced, the need to separately include these deformation uncertainties in the treatment margins may be limited.

## 1 INTRODUCTION

For gastric cancer, with a 5‐year survival of 20%–40%,^1^ gastrectomy remains the main curative treatment. Addition of chemo‐ and/or radiotherapy (RT) has been shown to improve outcome and due to severe toxicity and associated poor treatment compliance,^2^ focus has started to shift from adjuvant^2^, ^3^, ^4^ to neo‐adjuvant treatment.^5^ In the currently ongoing CRITICS‐II trial (45 Gy in 25 fractions; internal to planning target volume margin: 10 mm), the clinical target volume for pre‐operative chemoradiotherapy includes the tumour, first draining lymph node stations, as well as the entire stomach.^5^ The stomach, however, displays considerable motion, displacement and deformation, making accurate irradiation challenging.

Inter‐fractional changes in size and shape, due to changes in gastrointestinal filling, can be large^6^ and may be managed using image‐guided adaptive RT.^7^ This can, for example, be done with a cone‐beam computed tomography (CBCT)‐guided library‐of‐plans approach^8^ or on‐line adaptive magnetic resonance imaging (MRI)‐guided RT.^9^, ^10^ As also *during* irradiation changes take place, such as respiratory‐induced motion and peristalsis, these intrafractional changes must be quantified, for example, to establish required RT treatment margins.

Various studies quantified respiratory‐induced stomach motion (using fluoroscopy, CT or 4D‐MRI), reporting amplitudes of approximately 0.5–4 cm,^11^, ^12^, ^13^, ^14^ consistent with amplitudes found for other upper abdominal organs.^13^, ^15^, ^16^

Yet intrafractional stomach deformation data has been limited. MRI is very well‐suited to image gastrointestinal volumes,^17^ due to the extensive number of acquisition options and reconstruction protocols, each offering their own specific imaging assets. However, many of the studies touching on the subject of gastric deformation focus on frequency and amplitude of contraction waves, often in 2D^11^, ^18^, ^19^, ^20^ and do not provide the 3D deformation characteristics required for radiation treatment planning. 3D data was obtained in a study using a 4D MRI reconstruction technique with respiratory‐induced motion correction that yields 3D volumes depicting different phases in the gastric cycle^21^; the study focused on tissue displacements of surrounding organs caused by gastric deformation, which were found to reach up to 10 mm. The technique, however, only depicts periodic motion, which means part of the deformations may not be represented.

The aim of our study is to assess intrafractional stomach deformation over the surface of the stomach. MRI imaging of healthy volunteers was used to create 3D probability descriptions representing likelihood of occupation by the stomach, from which local deformation distributions were determined. Deformation was compared with respiratory‐induced displacement to assess potential effect on gastric cancer treatment planning.

## 2 METHODS

### 2.1 Imaging data

Eighteen healthy volunteers (young adults in their 20s and 30s; 10 male, eight female) were scanned in supine position on the Elekta Unity MR‐linac (Elekta AB, Stockholm, Sweden); no eating/drinking instructions were given; scan sessions were not scheduled at specific times during the day. Written informed consent was obtained from all volunteers on a medical ethics committee‐approved study protocol (NL62311.031.17). Volunteers are referred to as V1–V18 (scanning order), even though V7 and V15 were the same female scanned twice several months apart.

To obtain 3D probability descriptions for the stomach, 3D MR imaging would be preferable, as it allows for direct translation into positional variation over the entire surface of the stomach. However, as peristalsis is observable on a time scale of seconds,^22^ conventional 3D imaging is not fast enough to acquire images at the required resolution. Therefore, we chose to use 2D imaging to obtain a 3D probability description from which the positional variation of the stomach can be obtained, as explained in detail in the following subsections.

A 2D T_2_‐weighted multi‐slice coronal turbo spin echo sequence was used for image acquisition (FA/TR/TE = 90°/289–336 ms/60 ms, 1.54–1.87 mm pixel size, 5 mm slice spacing). The stomach was imaged in 27 (V16) or 25 slices in an interleaved fashion, repeated 120 times; total time 15–16 min. In 13 scans, the anterior side of the stomach was not completely captured due to too small a field of view. All analyses were performed in MATLAB 2021a, unless stated otherwise.

### 2.2 Auto‐segmentation

**FIGURE 1:**
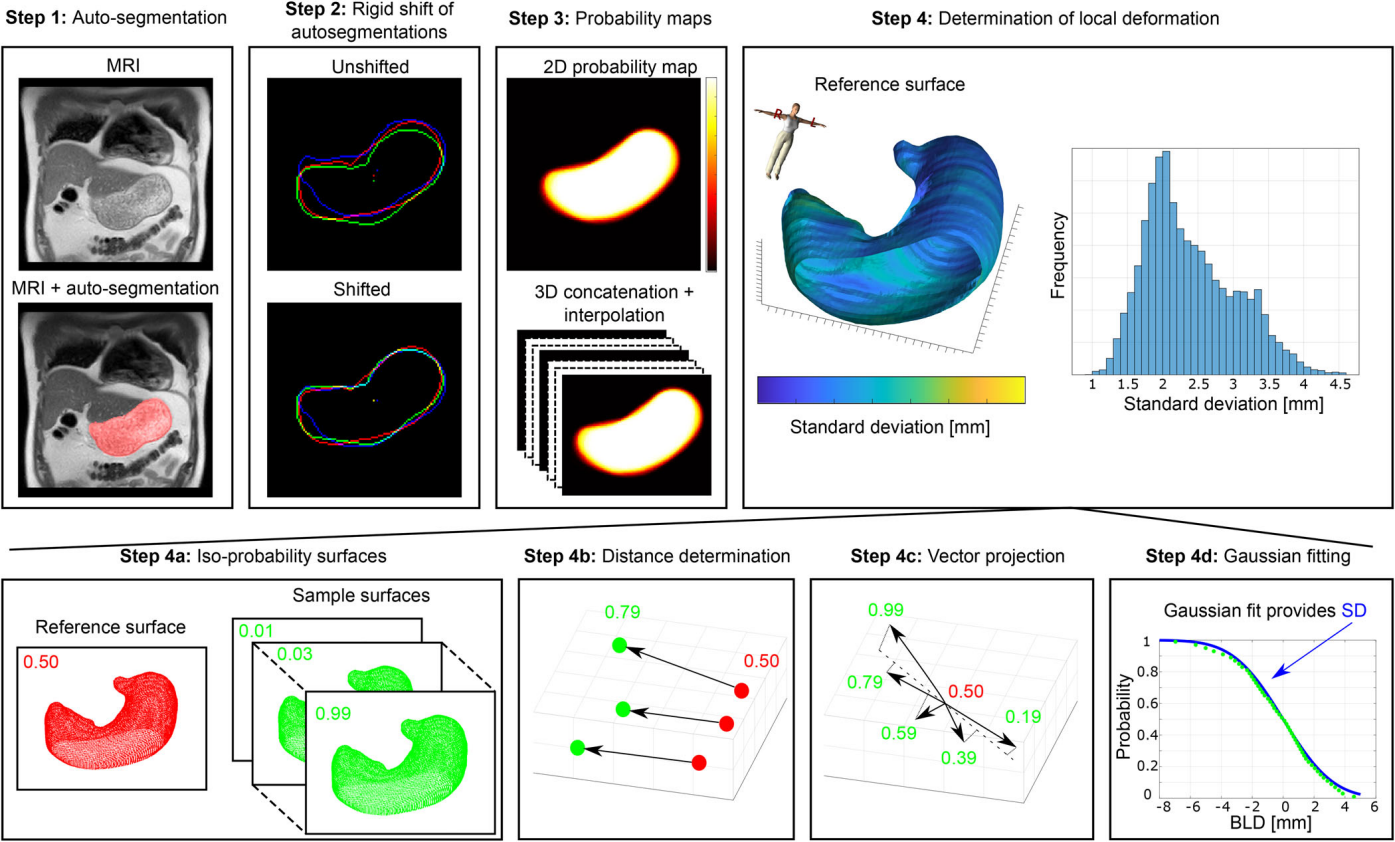
Schematic overview of local position variation determination. Stomach auto‐segmentations were obtained for all images of coronal MRI scans of 18 volunteers (step 1). These auto‐segmentations were rigidly shifted in superior‐inferior (SI) direction, based on centre of mass position, to separate out respiratory‐induced displacements (step 2). Then, for each slice a 2D probability map (PM) was created; these were concatenated in anterior‐posterior direction, and interpolated slices were inserted, to create 3D PMs (step 3). From these 3D PMs, iso‐probability surfaces (isosurfaces) were created (step 4a). For each point on the reference surface (i.e., 0.50‐isosurface), distances (3D bidirectional local distance, BLD) to 50 sample isosurfaces were determined (step 4b). For each point, these distance vectors were projected onto the mean distance vector (step 4c), yielding a probability‐distance dataset. By fitting a Gaussian cumulative distribution function to this dataset (step 4d), the local position variation, expressed as the standard deviation (SD), was obtained for each point on the reference surface. These SDs were represented as colour projections on the reference isosurface and as histograms.

The stomach was manually segmented (TD) in all slices of 10 of the 120 repetitions for all scans using ITK‐SNAP version 3.8,^23^, ^24^ yielding binary 2D images (i.e., pixels belonging to the stomach are 1, all others are 0). Using these manual segmentations, a deep‐learning neural network was trained to automatically segment the stomach (Figure 1, step 1). The network was based on a previously described U‐net architecture^25^; for details see the Supporting Information.

Performance of the model was validated by dividing the data into a training set (eight out of 10 available repetitions for each volunteer) and a test set (remaining two repetitions). For comparison, obtained auto‐segmentations and manual segmentations of the test set (904 images) were converted to contours; a contour consisted of all segmented pixels on the edge of the segmentation. Subsequently, the 2D bidirectional local distance (BLD)^26^ was used to determine the distance between manual and auto‐segmentation for each manual contour pixel, with positive distances indicating the auto‐segmentation contour was outside of the manual contour. While other metrics such as the Dice similarity coefficient and the Hausdorff distance are global measures yielding a single value per segmentation, the BLD provides for each segmentation a distribution of local distances, allowing for a deeper statistical analysis of the auto‐segmentation performance. In addition, the BLD provides a distance measure even for surfaces/contours with a high degree of curvature. Due to the curved shape of the stomach combined with 2D imaging, segmentation may yield multiple objects (i.e., separate parts of the stomach) and thus multiple contours in a single 2D image; only images with an equal number of manual and auto‐segmentation contours were included in the validation.

After validation, we trained the network again with the same parameters using all manual segmentations, yielding auto‐segmentations for all 120 repetitions.

### 2.3 Respiratory‐induced displacement

To separate respiratory‐induced displacement from deformation, a rigid shift was applied to the auto‐segmentations (Figure 1, step 2). For this, the centre of mass (CoM) of every auto‐segmentation was determined, along with the mean intra‐slice CoM. The auto‐segmentations were shifted in superior‐inferior (SI) direction (the largest respiratory‐induced motion component), so that the SI position of the CoM was equal to the SI position of the mean intra‐slice CoM (i.e., the CoM in the same slice averaged over all 120 repetitions). Due to its curved shape, out‐of‐plane motion of the stomach could lead, within a slice, to large SI CoM shifts not representative for respiration‐induced SI motion. To partially account for this, the mean intra‐slice CoM was determined separately for subgroups of images with equal number of segmented objects; when a subgroup contained <5 images, it was merged with the largest subgroup of that slice.

To assess respiratory‐induced motion, for each volunteer the distribution of applied CoM shifts was analysed and standard deviations (SD_resp_) reported.

With the respiratory‐induced SI displacement removed, we quantified local gastric deformation as SD_deform_, the SD of local position variation of the stomach (Figure 1, steps 3–4), as detailed in the following sections.

### 2.4 Iso‐probability surfaces

For each volunteer, 2D probability maps (PMs) were created for each slice by summing all auto‐segmentations and dividing the result by the number of repetitions (i.e., 120). These PMs were concatenated in the anterior‐posterior (AP) dimension to obtain a 3D PM describing the probability of that position being occupied by (a section of) the stomach (Figure 1, step 3). Empty slices were removed and, as the slice spacing (AP) was considerably larger than the pixel size [left‐right (LR) and SI], two additional linearly interpolated slices were inserted in between all remaining slices of the 2D PMs.

A marching cubes algorithm (MATLAB 2021a *isosurface* function) was used to generate iso‐probability surfaces from the 3D PMs (Figure 1, step 4a). The comparatively large slice distance in AP yields large steps in sampled probability in that direction. Consequently, the marching cubes algorithm can yield unrealistic estimates for the location of an isosurface on the anterior and posterior end of the stomach. This is exacerbated when too small a field of view results in missing data yielding higher probability values at the edge of the 3D PM. Therefore, for both the posterior and anterior side, an isosurface was left open at the edge of the 3D PM (Figure 1, step 4). For each volunteer, 51 isosurfaces were generated from the 3D PMs, for isovalues P_Occ_ ranging from P_Occ_ = 0.01–0.99 in steps of 0.02 and isovalue P_Occ_ = 0.50 (Figure 1, step 4a), with P_Occ_ indicating probability of that voxel being occupied by the stomach.

### 2.5 Local position variation

The 0.50‐isosurface was used as reference. For each point of this reference surface, the local distance (three‐dimensional BLD) to each isosurface was calculated (Figure 1, step 4b). The resulting 50 vectors were projected onto their mean (Figure 1, step 4c). The magnitudes of the projected vectors were determined, yielding a distance for each of the 50 probabilities, with negative distances for isovalues > 0.50.

For each point on the reference isosurface, a Gaussian cumulative distribution was fitted to its 50 probability‐distance data points to obtain an estimate for the local SD_deform_ (Figure 1, step 4d). The inserted interpolated slices increase the point density of the isosurfaces and therefore the accuracy of the distance measurements. When surfaces are locally very strongly curved, the spread in direction of BLD vectors can be very large, which can lead to a poor goodness of fit. Therefore, any obtained SD_deform_ with R^2^ < 0.8 was removed before further analysis.

Color‐coded projection of SD_deform_ onto the reference surface provided insight into the location of deformation. For each volunteer, we determined the median and 98^th^ percentile of SD_deform_ and compared these with SD_resp_ (paired Wilcoxon signed‐rank test, α = 0.05).

### 2.6 Method validation

The presented method of obtaining local deformation in 3D from 2D segmented images was validated with artificial datasets consisting of 120 concentric spheres, generated with normally distributed radii with fixed mean (60 mm) and certain input SD (1–6 mm); voxel size was comparable to voxel sizes of the volunteer data sets. After application of the method as described above, the obtained local SDs were compared with the input SD to assess the error. Details are given in the Supporting Information.

## 3 RESULTS

**FIGURE 2:**
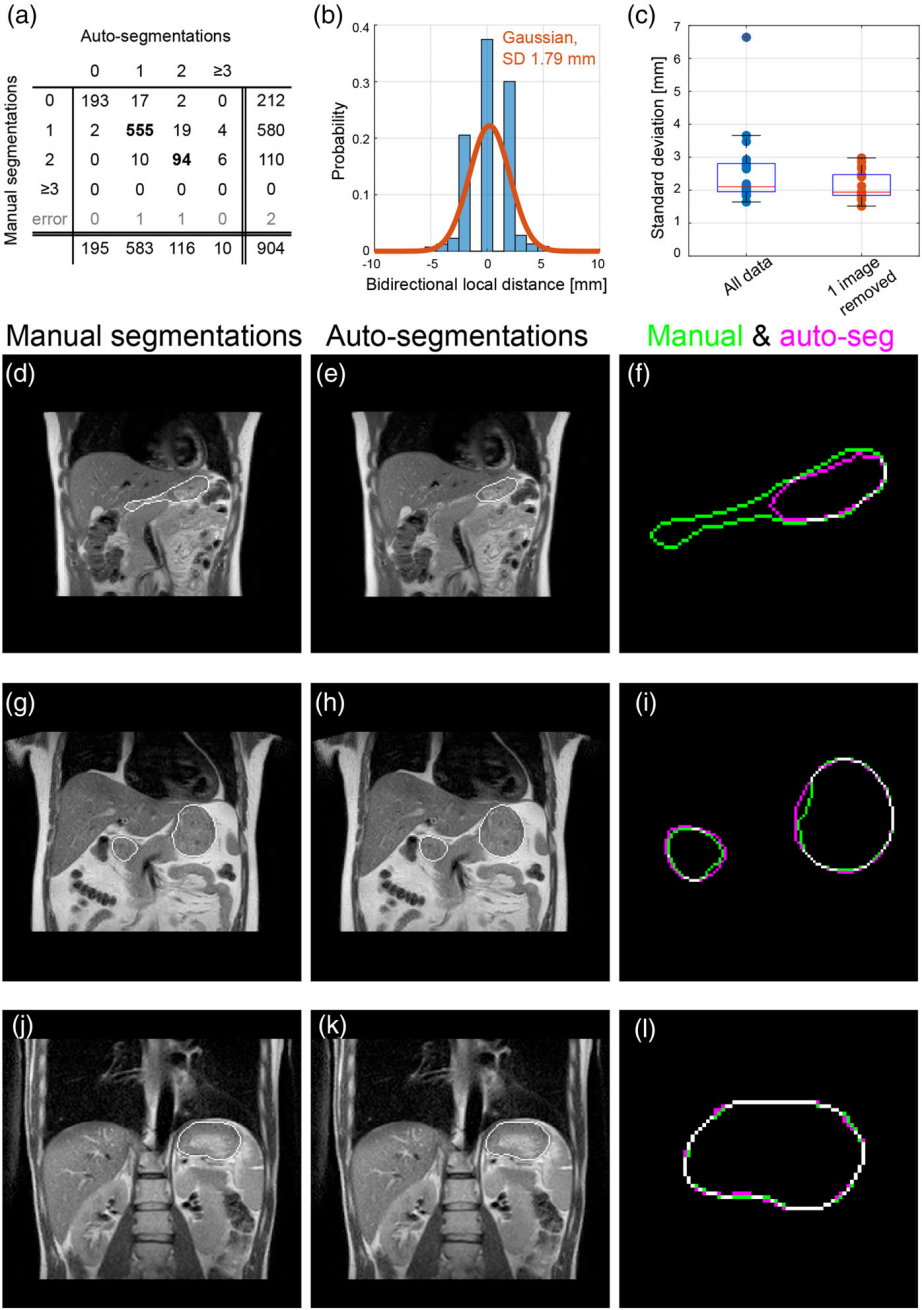
Auto‐segmentation validation. (a) Confusion matrix listing the number of contours identified by manual and auto‐segmentation, for each of the 904 images used; only the images for which the numbers agreed (in bold, 649 images in total) were used for verification. Two images were excluded, because due to an error the manual segmentations did not belong to the associated image. (b) Histogram of measured distances (2D bidirectional local distance) between manual and auto‐segmentation contours, for each pixel of the manual contours. For the 98% smallest distance range the standard deviation (SD) was 1.79 mm (corresponding Gaussian in orange). (c) Boxplot of SDs of measured distances per volunteer, for complete datasets (left) and when for each volunteer the one image with largest SD was removed (right). (d–l) Examples of manual (green) and auto‐segmentations (magenta) compared, showing large differences, for example due to contrast decrease from partial volume effects (d–f; in‐slice SD 20.9 mm), intermediate differences (g–i; 1.8 mm) and small differences (j–l; 0.8 mm); contour in white when contours coincide.

Auto‐segmentation performed well. For the 649 test set images with equal numbers of contours (Figure 2a), overall mean distance between manual and auto‐segmentation contour was 0.17 mm, indicating no bias towards over‐ or under‐segmentation. Although large errors did occur, 98% of pixels in the manual contours were within −5.9 to 7.0 mm and yielded an SD of 1.79 mm (Figure 2b). For some volunteers, the obtained SD strongly depended on an individual image with large errors; when removing for each volunteer the one image with largest SD, SDs ranged from 1.5 to 3.0 mm (median, 1.9 mm; Figure 2c). The large differences in segmentation mostly occurred due to partial volume effects decreasing contrast, which was especially apparent in slices where the stomach had a large out‐of‐plane curvature (Figure 2d–i), as well as due to gastro‐intestinal gas and a lack of anatomical boundary at the oesophagus. Overall, for 69% of pixels the error was one pixel or less (i.e., ≤1.87 mm, Figure 2j–l).

When validating our method using the artificial datasets, we found that 79% of the obtained SDs were within −10% and +20% of the input SD (Supporting Information). Results varied depending on input SD, due to the interplay between voxel size and input SD. As a result of the voxel size being larger in AP than in SI and LR, SD overestimations were largest at the anterior and posterior sides of the spheres.

**FIGURE 3:**
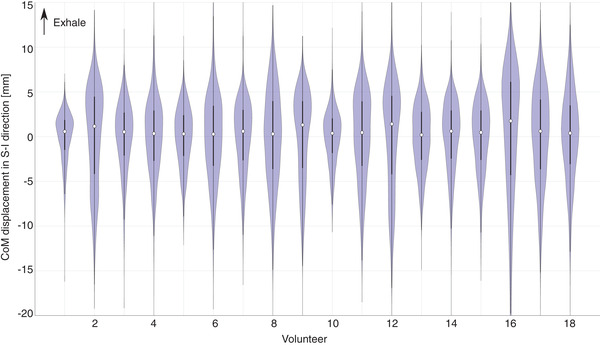
Respiratory‐induced displacement: applied shifts along the superior‐inferior (SI) axis, based on CoM of auto‐segmentations. Per volunteer, a violin plot indicates probability density, median (open circle) and inter‐quartile range (thick line). Positive shifts are associated with exhale. For visibility, y‐axis range is limited; data points outside of shown range are < 0.5% per volunteer, except for V16 (3.4%).

Respiratory‐induced motion varied between volunteers, with SD_resp_ between 2.7 and 8.8 mm (Figure 3, Table 1).

**TABLE 1:** Deformation (median and 98^th^ percentile of SD_deform_) and respiratory‐induced SI displacement (SD_resp_): Median and range over all volunteers. Median of SD_deform_ was smaller than SD_resp_ (*p* = 7.6e−06; paired Wilcoxon signed‐rank test); 98^th^ percentile of SD_deform_ did not significantly differ from SD_resp_ (*p* = 0.130).

	Deformation		Displacement of CoM in SI
	Median of SD_deform_	98^th^ percentile of SD_deform_	SD_resp_
Median [mm]	2.4	5.0	4.6
Range (min–max) [mm]	2.0–2.9	3.2–7.3	2.7–8.8

For the 18 volunteers, the volume of the reference isosurfaces ranged from 118 to 900 ml (median, 308 ml). Fewer than 0.4% of data points were removed due to poor goodness of fit (maximum per volunteer, 2.7%).

**FIGURE 4:**
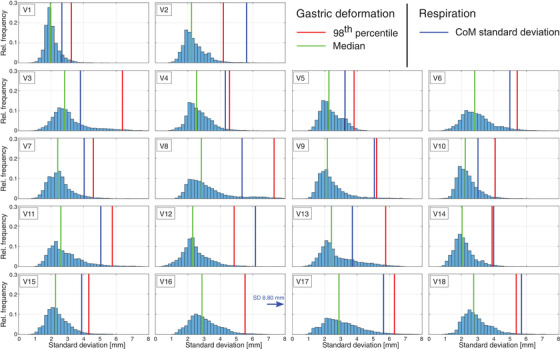
Histograms of all measured SD_deform_ of gastric deformation, with median (green line) and 98^th^ percentile (red) indicated; points with R^2^ < 0.8 were not included. For comparison, SD_resp_ of respiratory‐induced SI displacement is shown (blue). Distributions differed between volunteers, ranging from limited (e.g., V1, V5, V14) to more pronounced deformation (e.g., V3, V8, V17).

**FIGURE 5:**
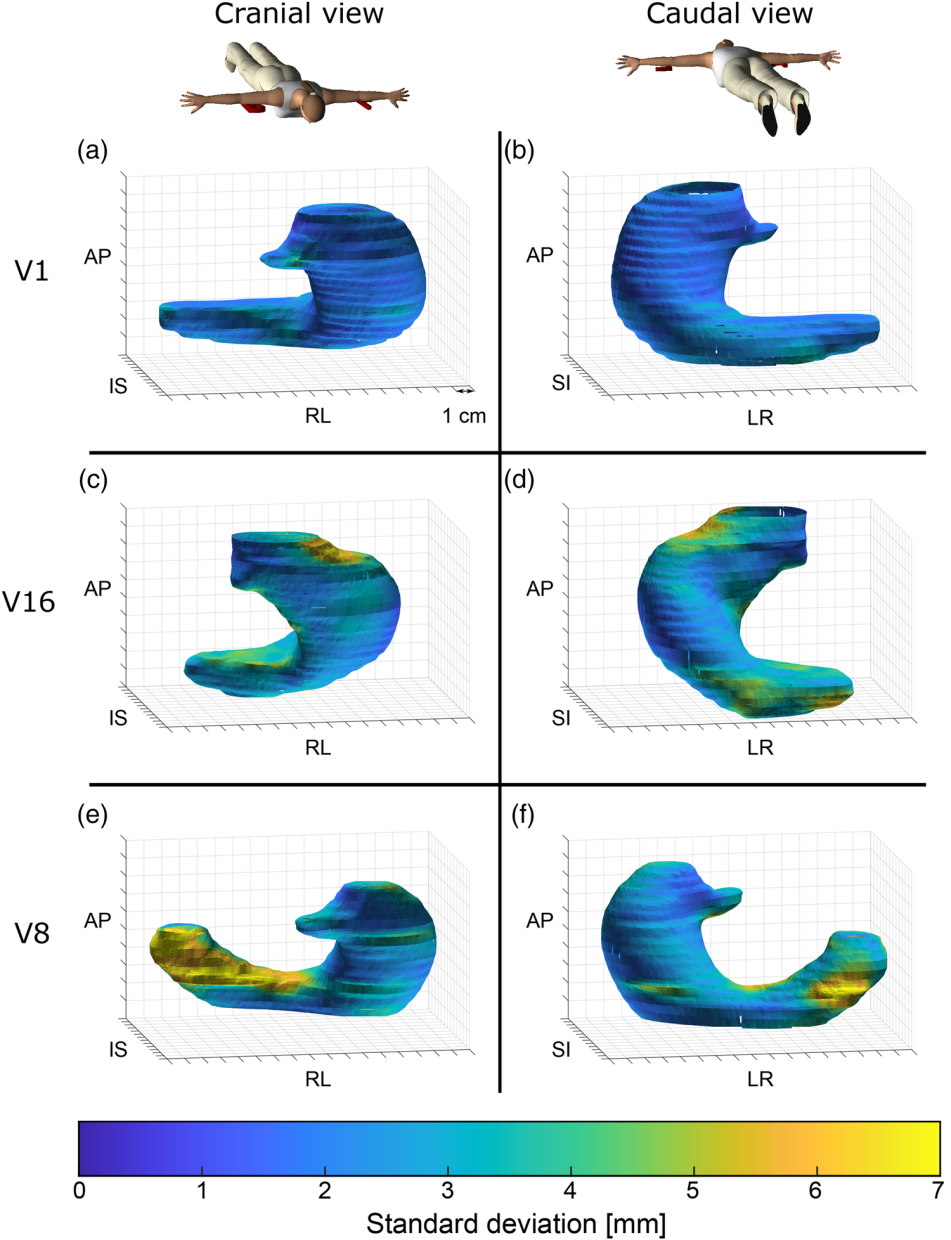
Examples of measured SD_deform_ of local position variation projected onto the reference isosurface, for three volunteers, in cranial (left) and caudal view (right). (a–b) Volunteer V1: small overall local deformation (98^th^ percentile SD_deform_ of 3.2 mm). (c–d) V16: moderate deformation, near fundus and antrum (98^th^ percentile SD_deform_ of 5.5 mm). (e–f) V8: considerable gastric deformation (98^th^ percentile SD_deform_ of 7.3 mm), mainly around the antrum.

Extent of deformation differed considerably between volunteers (Figure 4, Table 1). Although no clear subgroups could be identified, limited magnitude of and variation in deformation were found for volunteers V1, V5 and V14, with median SD_deform_ 2.0–2.3 mm and 98^th^ percentile SD_deform_ < 3.9 mm. Figure 5a,b shows, as an example, the distribution for V1; the very localized high local deformity with high SD_deform_ near the oesophagus (Figure 5a) is likely due to a segmentation error. Many volunteers displayed moderate deformation, such as for example volunteer V16, with deformation mostly around the pyloric canal and caudal section of the greater curvature (Figure 5c,d). For volunteers V3, V8 and V17, large deformations were seen, with a median SD_deform_ of 2.8–2.9 mm and 98^th^ percentile SD_deform_ > 6.2 mm. Regions of large deformation were most often near the antrum and pyloric canal, as seen for instance in volunteer V8 (Figure 5e,f). Overall, displacement due to respiration was larger than deformation, but comparable to the 98^th^ percentile of deformation (Figure 4, Table 1). Both median and 98^th^ percentile SD_deform_ did not correlate with the volume of the reference isosurface (absolute Spearman rank correlation coefficient |ρ| < 0.3, *p* > 0.29).

## 4 DISCUSSION

In this work, we used MRI scans of healthy volunteers to quantify local intrafractional gastric deformations using 3D probability descriptions of the stomach and compare these deformations with assessed respiratory‐induced stomach displacement in SI direction.

Overall, deformations (with range of median SD_deform_ 2.0–2.9 mm) were smaller than respiratory‐induced displacement, but locally deformations with SD_deform_ > 6 mm were seen. Regions of large deformation were mostly found in the antrum and pyloric region, consistent with gastric peristaltic contractions mostly taking place in the distal stomach.^22^

We used a deep‐learning neural network to segment the stomach on the MRIs. Validation showed that our auto‐segmentation overall performed well, with 69% of pixels a single pixel or less away from the manual segmentations. Most of the large segmentation errors (including slices with a difference in number of segmented objects, which were excluded from our performance analysis) were due to segmentation being done in 2D; a small (e.g., slice distance) difference in 3D might be depicted and/or segmented as a large difference when imaging in 2D. Therefore, for many of these large segmentation errors, the effect on the obtained 3D isosurfaces would have been limited. In addition, the large errors were only found in a few images, further limiting the effect on the isosurfaces and thus on local SD_deform_, given that SD_deform_ is obtained from a fit to 50 probability‐position datapoints. The error of auto‐segmentation was relatively small compared to our measured deformations of interest, with the SD of 1.8 mm < median SD_deform_ for each volunteer.

Errors were also introduced when filtering out respiratory‐induced motion. Shifts were only applied in SI, even though motion also takes place in AP and RL directions, albeit to a lesser extent. Wysocka et al., for example, reported median gastric respiratory‐induced motion (difference between end‐inspiration and end‐expiration breath‐holding scans) of 16.4, 8.8 and 1.7 mm in SI, AP and RL, respectively^13^; with the motion in RL so much smaller than in SI, we chose to only apply shifts in SI. These shift errors (in SI and RL) potentially contributed to the measured SD_deform_; however, this contribution can be expected to be small, as errors in SI and RL shift will be considerably smaller than the median SD_resp_ (in SI) of 4.6 mm (Table 1). In addition, errors due to large shape changes as a result of out‐of‐plane motion (AP) were only partially accounted for by analysing the CoM positions per group based on number of contours present. However, larger errors likely only occurred in a limited number of images, with minimal effect on SD_deform_, as the local SD_deform_ is based on a fit to 50 probability‐position datapoints.

When using the applied SI shift to obtain SD_resp_, our measure for respiratory‐induced motion, several issues should be considered. Due to the domed shape of the diaphragm, the extent of respiratory‐induced displacement of upper abdominal organs depends on the AP position^27^; in the current study, however, we summarized the displacement by combining data over all MRI slices. Even though we only determined respiratory‐induced displacements in SI, our results were comparable to those found by others.^13^ Respiratory‐induced motion is typically skewed towards the end‐exhale position. However, as the effect of such asymmetry on dose distributions and treatment margins has been shown to be minimal,^28^ our approach of reporting SD of CoM shifts without accounting for skewedness is justified.

Our study regarded young healthy volunteers; gastric deformation may be different in patients. However, a study in patients found mean gastric deformations of 5 mm (maximum 12 mm),^21^ comparable to our results. Also, unlike in clinical practice, the volunteers did not receive eating instructions prior to image acquisition. Nevertheless, even though the reference isosurface volume may not necessarily be identical to true stomach volume, the range of our reference volumes (118–900 cm^3^) was comparable to ranges of stomach volumes found in patient studies (e.g., 90–1100 cm^3^ ^21^ and 160–1362 cm^3^ ^6^). However, it should be noted that our cohort was, with 17 individuals, limited in size. In addition, we measured deformation of stomach rather than clinical target volume, which also includes lymph nodes.

Our method to determine local deformation was validated using simulated data. The largest errors in obtained SD_deform_ were found at the anterior and posterior ends, due to the relatively large slice spacing of 5 mm. As isosurface positions were unreliable when having to extrapolate beyond the edge of the 3D PM, we left the isosurfaces open at the posterior and anterior ends in our analysis of volunteer data. The simulations showed this had little effect on the obtained SD_deform_ for the remaining reference isosurface (Supplemental Figure A1).

However, with the reference surface fully closed, the obtained SD_deform_ at the ends was likely less accurate; measured local deformations at the ends appeared in line with deformations found in nearby regions, though. If more accurate measurements are required for the anterior and posterior sides, different or additional scanning directions and/or techniques are needed. Overall, with an error between −10% and +20% for 79% of datapoints, our method performed well, indicating a sufficient accuracy of our results in our volunteer study.

For some volunteers, no local deformation was obtained for the anterior and/or posterior sides, as the reference surface was open at these locations due to too short a scanning volume; respiratory motion moves the stomach in and out of coronal planes. Scanning a large volume would have prevented this. Obtaining more accurate data on anterior and posterior sides for all volunteers could be done by expanding the analysis with sagittal imaging.

Assessing the potential impact on an estimate of a margin M, we used M = 2.5Σ + 0.7σ,^29^ with systematic errors Σ for delineation (3 mm) and setup (1 mm), and random errors σ for respiration and deformation, assuming image‐guided RT. For the SI direction, when applying 4.6 mm for respiration, impact of deformation is limited: a σ_deform_ of 5 mm (Table 1, median 98^th^ percentile of SD_deform_) rather than 0 mm, yields a margin of 12.7 instead of 11.1 mm. However, in the AP and LR directions, respiratory‐induced motion is typically smaller than in SI.^13^ Respiratory‐induced motion may be reduced (e.g., by breath holding)^30^ or dealt with using gating; for smaller respiratory‐induced motion, the impact of deformation on margins will be larger. Full potential dosimetric effects of gastric deformation in pre‐operative gastric cancer irradiation would have to be investigated in a dosimetric study.

With adaptive RT, it becomes possible to account for changes in anatomy between treatment planning and delivery as well as over the course of RT.^7^ Day‐to‐day changes in stomach size and shape due to changes in filling (excluded from the above margin assessment) can be considerable (on the scale of centimeters^6^, ^12^) and should be managed using adaptive RT, such as a library‐of‐plans approach,^8^ rather than be included in a margin. Novel devices such as the MR‐linac allow for imaging during treatment delivery and consequently for margin decrease. With tighter margins, local deformation may have to be included in the treatment plan.

## 5 CONCLUSION

Locally, large gastric deformations can take place. Overall, however, the extent of these deformations is limited in comparison to respiratory‐induced motion. Whether intrafractional gastric deformation should be taken into account in determining treatment margins will depend on the magnitude of other uncertainties; for treatment in free breathing, likely the effect of including gastric deformation uncertainties on calculated margins is limited.

## AUTHOR CONTRIBUTIONS

Theo Driever, Jan‐Jakob Sonke and Astrid van der Horst contributed to the conception, design and analysis of the study. Figure preparation and most analyses were performed by Theo Driever. First draft of the manuscript was written by Theo Driever and Astrid van der Horst; all other authors commented on and edited the manuscript. All authors contributed to the study.

## CONFLICT OF INTEREST

The Department of Radiation Oncology of the Netherlands Cancer Institute receives license fees from Elekta AB (Sweden) for cone beam CT‐guided software. A. Bel is involved in several projects sponsored by Elekta AB (Stockholm, Sweden), Varian (Palo Alto, CA, USA) and Medlogix (Rome, Italy).

## Supporting information

Supporting Information
