# Supplementary material for: Quantifying intrafractional gastric motion using auto‐segmentation on MRI: Deformation and respiratory‐induced displacement compared
Source: J Appl Clin Med Phys. 2022 Dec 24;24(4):e13864. doi: 10.1002/acm2.13864 (PMC10113698; doi:10.1002/acm2.13864)
Supplement: Supplementary file 1 — Supporting Information [file ACM2-24-e13864-s001.pdf]

Appendix A. Supplementary Materials

Supplementary material belonging to **Quantifying intrafractional gastric motion using auto-segmentation on MRI: deformation and respiratory-induced displacement compared**

by Theo Driever, Maarten CCM Hulshof, Arjan Bel, Jan-Jakob Sonke and Astrid van der Horst

Supplementary material I: Details on the neural network used for auto-segmentation

We used the 2D variant of the U-Net presented in [**Nikolov2018**] to predict segmentation maps for the stomach. The 2D architecture is very similar to the original U-Net [**Ronneberger2015**]. The key differences are the use of residual connections and an extended bottleneck. Further details on the network are provided below.

Encoder

The encoder consists of five down-sampling blocks. Each block contains two consecutive convolutional layers using filters of size 3×3. Each layer is followed by a group normalization, using four groups, and a ReLu activation. The input to the convolutional layers are padded with zeros to preserve the spatial dimensions. A residual connection is used to add the input of the block to the (activated) output of the second convolutional layer. To accomplish this, the number of channels of the input is aligned with the desired number of channels by using a 1×1 convolution. Finally, the spatial dimensions are reduced (halved) by using an average-pooling layer of size 2×2. The number of filters used in the five blocks, from top to bottom, are 32, 64, 128, 128 and 256, respectively. The output of the encoder is a feature map of size 256×8×8.

Bottleneck

The encoder is followed by a bottleneck, which consists of four fully connected layers. The first layer maps the output of the encoder to a 512-dimensional vector followed by a ReLu activation. The next two layers map from R512 to R512, where each layer is followed by a ReLu activation and a residual connection. The fourth layer maps the 512-dimensional feature vector back to a feature map of size 256×8×8.

Decoder

The bottleneck is followed by a decoder, which consists of five upsampling blocks. Each block starts with doubling the spatial dimensions of the input using bilinear interpolation. Subsequently, the upsampled input is concatenated with the feature map from the encoder path that corresponds to the same spatial level (a skip-connection) and is followed by a ReLu activation. The remainder of the block consists of two convolutional layers as before, using filters of size 3×3, group normalization and ReLu activation, followed by a residual connection. The number of filters used in the decoder, from bottom to top, is 256, 128, 64, 64, 64, respectively. The output of the decoder is a feature map of size 64×256×256.

Final layer (segmentation map)

The output of the decoder is reduced to a feature map of size 256×256 using a 1×1 convolution. Finally, a sigmoid activation map is used to construct the desired probability map.

Augmentation and optimization

Extensive data augmentation (rotation, shift, scale, shear, elastic deformation, flip, Gaussian noise, blurring and gamma) was used to reduce the generalization error. The model was optimized by minimizing the weighted focal cross-entropy [**Lin2020**]. The gradient descent steps were based on the pixels with a relatively large loss by setting the exponent of the focal weight to two. An L^2-penalty with weight 10^{-5} and a dropout rate of 0.2 were used for regularization.

References

[**Nikolov2018**] Nikolov S, Blackwell S, Mendes R, De Fauw J, Meyer C, Hughes C, et al. Deep learning to achieve clinically applicable segmentation of head and neck anatomy for radiotherapy.
https://arxiv.org/abs/1890.04430

[**Ronneberger2015**] Ronneberger O, Fischer P, Brox T (2015) U-Net: Convolutional Networks for Biomedical Image Segmentation. In: Navab N, Hornegger J, Wells W, Frangi A (eds.) Medical Image Computing and Computer-Assisted Intervention – MICCAI 2015. MICCAI 2015. pp. 234–241 Lecture Notes in Computer Science, vol 9351. Springer, Cham.
https://doi.org/10.1007/978-3-319-24574-4_28

[**Lin2020**] Lin T, Goyal P, Girshick R, He K, Dollár P. Focal loss for dense object detection. IEEE Transactions on Pattern Analysis and Machine Intelligence 2020;42:318–327.
https://doi.org/10.1109/TPAMI.2018.2858826

Supplementary material II: Method validation

The presented method to assess local position variation was validated with artificial datasets consisting of concentric spheres.

For a single validation iteration, **120** concentric spheres were generated with radii drawn from a normal distribution. The origin was located at the centre of a voxel. The mean radius of the spheres was **60mm**, similar to a typical radius of a filled stomach. When the centre of a voxel fell on or within the sphere's radius, the voxel value was set to one, all other voxels were zero; voxel size was **$1.67 \times 1.67 \times 5.00mm^3$** (LR×SI×AP).

The 2D slices of the dataset, each effectively containing a 2D pixelated cross-section of a sphere, were used to create 2D PMs. Subsequently, the method as described in the manuscript was applied: two linearly interpolated slices were added in AP; 3D PMs and iso-probability surfaces (isosurfaces) were created and distances between isosurfaces and the reference surface were determined; using Gaussian cumulative distribution fits, the standard deviation (SD) of local deformation was obtained for each point on the reference 0.50-isosurface. Data for which the fit yielded $R^2<0.80$ were disregarded.

Due to the limited sample size of 120 radii, the actual SD of each artificial dataset is not equal to the input SD. Therefore, in this Appendix, we use the normalized SD, which is obtained by dividing the SDs from the Gaussian cumulative distribution fit by the *actual* SD, i.e. the calculated SD of the input radii.

1. Effect of open anterior and posterior sides

Because the distance between slices (AP, 5mm) is larger than the in-plane pixel size ($1.67 \times 1.67mm^2$), the error depends on the angle between the surface normal and the image plane (i.e. the slice). As a consequence, the marching cubes algorithm can yield unrealistic estimates for the location of an isosurface in the AP direction, resulting in large errors on anterior and posterior sides. Therefore, we left the anterior and posterior sides open in our analyses of the volunteer data.

To investigate the effect of this approach on the estimate of error in our SD determination, we removed all empty slices from our validation data set and subsequently compared for a single dataset (mean radius 60mm; SD 3.05mm) three situations:
(a) **zero** additional slices removed (i.e. full dataset);
(b) **three** additional slices removed on either side;
(c) **six** additional slices removed on either side.

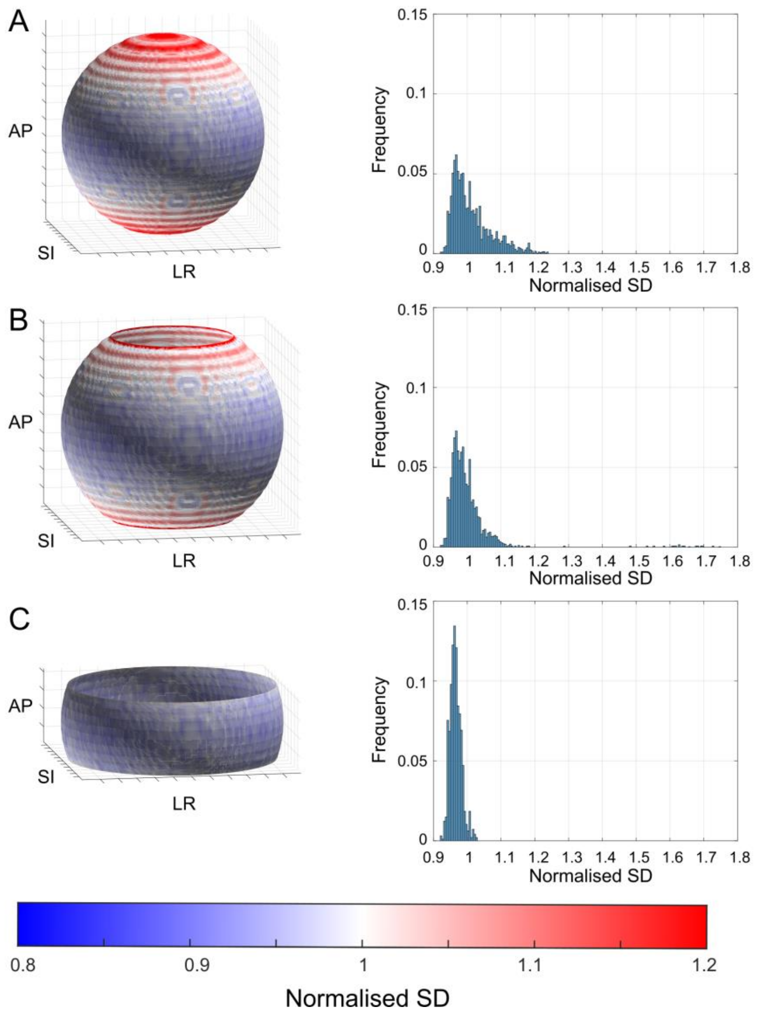

Figure A1. *Colour projections and histograms of obtained normalized SDs for (a)* ***zero*** *additional slices removed, (b)* ***three*** *additional slices removed on either side and (c)* ***six*** *additional slices removed on either side. Input SD was 3.05mm.*

2. Interplay effect between sphere radii and pixel size

Due to the interplay between sphere radius and voxel size, the exact outcome for an iteration depends on the input SD. To illustrate the effect, we ran three iterations with slightly different input SD:
(a) 4.06031mm; (b) 4.07736mm; (c) 4.07927mm.

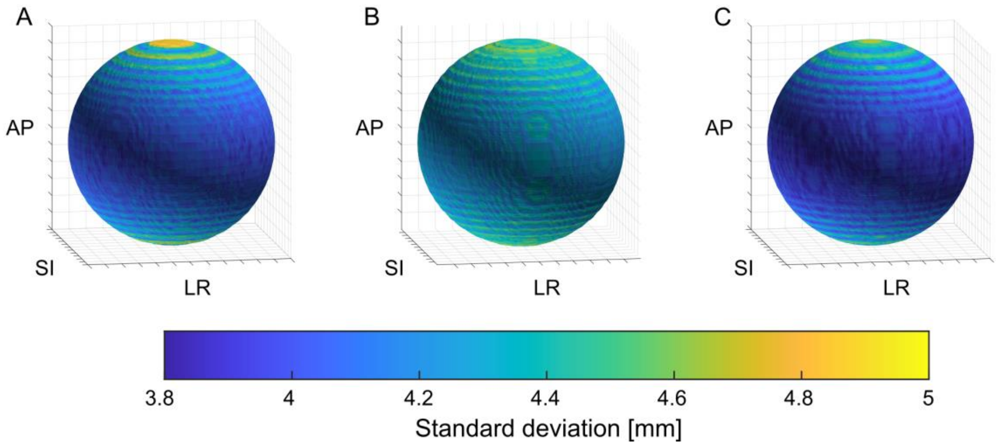

Figure A2. *Colour projections for three iterations with slightly different input SD. Due to the interplay effect between sphere radius and voxel size, outcome can differ.*

3. Overall error estimate: 100 iterations

As the exact outcome for an iteration depends on the input SD, we ran **100** iterations, with the input SD of the radii randomly chosen from a uniform distribution with range 1-6mm.

For the situation with 3 slices on each side removed (most representative for our volunteer data analysis), we found that **79%** of points had an error within -10% and +20%.

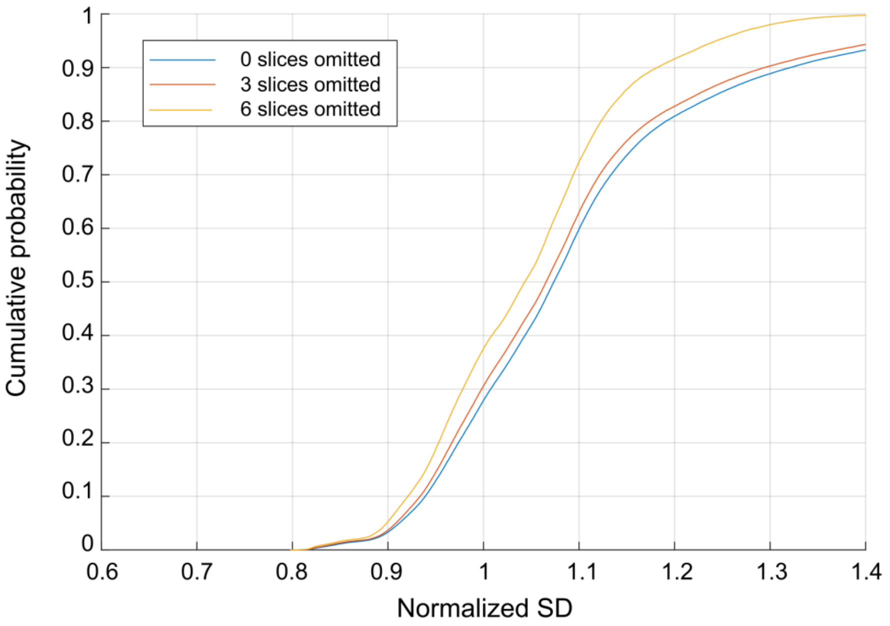

Figure A3. *Cumulative histograms of obtained normalized SDs for 100 iterations each, for (a)* ***zero*** *additional slices removed, (b)* ***three*** *additional slices removed on either side and (c)* ***six*** *additional slices removed on either side. Input SD varied between*
